# A comprehensive guide to BCI-based stroke neurorehabilitation interventions

**DOI:** 10.1016/j.mex.2023.102452

**Published:** 2023-10-18

**Authors:** Jessica Cantillo-Negrete, Ruben I. Carino-Escobar, Emmanuel Ortega-Robles, Oscar Arias-Carrión

**Affiliations:** a División de Investigación en Neurociencias Clínica, Instituto Nacional de Rehabilitación Luis Guillermo Ibarra, Mexico City, NM 14389, Mexico; b Unidad de Trastornos del Movimiento y Sueño, Hospital General Dr. Manuel Gea González, Mexico City 14080, Mexico

**Keywords:** Brain-machine interfaces, Hemiparesis, Transcranial magnetic stimulation, Robotics, Neurotechnology, A comprehensive guide for conducting BCI-Based therapy in stroke rehabilitation

## Abstract

Brain-Computer Interfaces (BCIs) offer the potential to facilitate neurorehabilitation in stroke patients by decoding user intentions from the central nervous system, thereby enabling control over external devices. Despite their promise, the diverse range of intervention parameters and technical challenges in clinical settings have hindered the accumulation of substantial evidence supporting the efficacy and effectiveness of BCIs in stroke rehabilitation. This article introduces a practical guide designed to navigate through these challenges in conducting BCI interventions for stroke rehabilitation. Applicable regardless of infrastructure and study design limitations, this guide acts as a comprehensive reference for executing BCI-based stroke interventions. Furthermore, it encapsulates insights gleaned from administering hundreds of BCI rehabilitation sessions to stroke patients.

• Presents a comprehensive methodology for implementing BCI-based upper extremity therapy in stroke patients.
• Provides detailed guidance on the number of sessions, trials, as well as the necessary hardware and software for effective intervention.

## Graphical abstract

**Figure:**
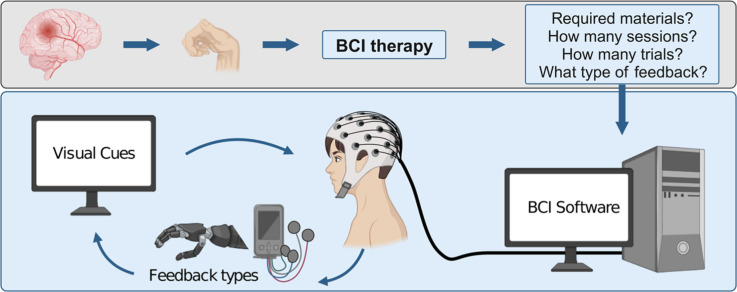

Specifications Table

**Table:** 

Subject area:	
More specific subject area:	Neurorehabilitation
Name of your method:	A comprehensive guide for conducting BCI-Based therapy in stroke rehabilitation
Name and reference of original method:	None
Resource availability:	Does not apply

## Method Details

Various devices, ranging from computer cursors to robots, can be controlled by systems capable of decoding information from the central nervous system and of translating this information into external commands [1]. These systems are known as Brain-Computer Interfaces (BCIs). A BCI consists of acquisition, processing, external device control, and feedback stages [1]. In the acquisition stage, raw data is obtained from the central nervous system, with electroencephalography (EEG) being the most commonly employed technique [2]. EEG records the brain's electrical activity and offers non-invasiveness and suitable time resolution for detecting users' intentions. The processing stage involves filtering signals, extracting features related to the user's intentions, classifying them, and sending them as command signals to an external device, which represents the final stage of a BCI. To control a BCI, a strategy or paradigm is necessary to code users' intentions based on information extracted from their central nervous system. One commonly used BCI paradigm is sensorimotor rhythms, which are patterns of electrical neural activity observed in the EEG, primarily in the alpha (8–13 Hz, particularly the mu rhythm) and beta (13–30 Hz) frequency bands of cortical activity [3]. This paradigm has the advantage of not relying on external stimuli. Motor imagery or motor intention (MI), referring to mentally practicing moving a limb without physically doing so, are often employed for decoding sensorimotor rhythms in BCI applications, as cortical activations elicited during such tasks resemble those elicited during actual movement execution [4]. These movement-related activations can be recorded using EEG in regions close to the central and parietal positions of the international 10–20 electrode positioning system, making them particularly relevant for applications related to neurological disease rehabilitation. Consequently, EEG-based BCI systems controlled using MI have shown potential for stroke neurorehabilitation [5].

Stroke is a sudden neurological deficit caused by the obstruction (ischemic) or rupture (hemorrhagic) of brain blood vessels, leading to death or disability. By 2019, it was estimated that 101 million individuals worldwide were living with stroke sequelae [6]. The prevalence of stroke is expected to rise globally due to the association between SARS-CoV-2 infection and an increased risk of ischemic stroke [7]. Hemiparesis, the partial paralysis of one side of the body, is a common sequela of stroke resulting from neurological damage within the corticospinal tract, impairing or inhibiting voluntary movement. Even with conventional treatment, most patients with a high degree of paresis experience incomplete recovery [8]. In the past decade, BCI systems have been explored as a complementary therapy for stroke neurorehabilitation, based on the hypothesis that augmented feedback can promote neuroplasticity, the primary recovery mechanism for hemiparesis. Numerous clinical trials have been conducted, with many reporting positive effects of BCI interventions on functional recovery of the upper or lower extremities [9,10]. However, the clinical effectiveness of BCI interventions for stroke rehabilitation still lacks robust evidence due to the challenges associated with implementing BCI technology in healthcare settings [10].

A crucial aspect of neurorehabilitation using Motor Imagery (MI)-based Brain-Computer Interface (BCI) systems is the choice of MI paradigm employed during the intervention. MI has been extensively utilized for BCI control by decoding user intentions from changes in the alpha and beta rhythms of the EEG, which can be identified through signal processing techniques. In stroke neurorehabilitation, MI of the affected limb is typically employed for BCI control [9,10]. However, this poses a significant challenge due to the substantial variability among potential BCI users, stemming from factors such as gender [11], spatial ability [12], BCI setups [13], [14], [15], among others. For addressing the complexity of decoding MI information from the EEG, signal processing techniques and machine learning algorithms have been employed, with the Common Spatial Patterns (CSP) algorithm being one of the most widely utilized for MI [16,17]. This algorithm filters each EEG channel using spatial information derived from all recorded channels to maximize logarithmic variance for one type of motor task and minimize it for another. An extension of this algorithm, known as Filter Bank Common Spatial Patterns (FBCSP), applies CSP on EEG signals filtered at different frequencies [17]. Subsequently, the filtered signals can be selected and classified using machine learning techniques, with Linear Discriminant Analysis (LDA) being frequently employed for this purpose [18].

Another critical aspect of BCI systems is the type of augmented feedback, which refers to the strategy employed to provide users with information regarding their performance or the decoding outcomes. Different types of feedback have been shown to elicit variations in cortical activations both in terms of the degree of activity and the regions involved [[13], [14], [15],^19^,^20^]. Feedback can be broadly classified as visual, auditory, or sensory. In stroke neurorehabilitation, sensory feedback is the most reported, although visual feedback, often combined with sensory feedback, has also been utilized [9]. This preference for sensory feedback is likely due to its potential to induce greater cortical activations, hypothesized to promote neuroplasticity in stroke patients [21]. Sensory feedback can be further categorized into robotic rehabilitation devices and neuromuscular electrical stimulation. Robotic rehabilitation devices have been extensively investigated in stroke neurorehabilitation studies and provide passive movement to the limbs of patients through an electromechanical system, making them a form of complementary therapy [22], [23], [24], [25]. It has been hypothesized that integrating BCI control into robotic rehabilitation devices can enhance the clinical effectiveness of interventions [26,27]. Neuromuscular electrical stimulation (NMES) involves the use of implanted or superficial electrodes that deliver a modulated electrical current within a frequency range of 12 Hz to 50 Hz, an amplitude of 0–100 mA, and a pulse width of 0–300 µs to induce muscle contraction [28]. Although fewer stroke intervention studies have utilized BCI with NMES as feedback compared to robotic rehabilitation devices, this type of stimulation can assist in the recovery of muscular tone and is itself a form of stroke therapy [28].

In this article, we present a comprehensive methodology for conducting upper extremity rehabilitation interventions of stroke patients using EEG-based BCI systems. This protocol serves as a valuable reference document for EEG-based BCI researchers, equipping them with the necessary knowledge and preparedness to tackle the challenges associated with performing BCI interventions in stroke populations. We address these challenges from engineering, neurological, and rehabilitation perspectives, ensuring a holistic understanding of the intervention process. The method is designed to be applicable to various BCI setups while considering the unique circumstances often encountered in the clinical setting of stroke patients. As such, it encompasses technical, physiological, and clinical considerations to provide a well-rounded approach. Additionally, we share insights and notes gathered from years of experience working with BCI protocols, including clinical trials focused on stroke rehabilitation.

## Materials

### Patient Setup

**Fig. 1:**
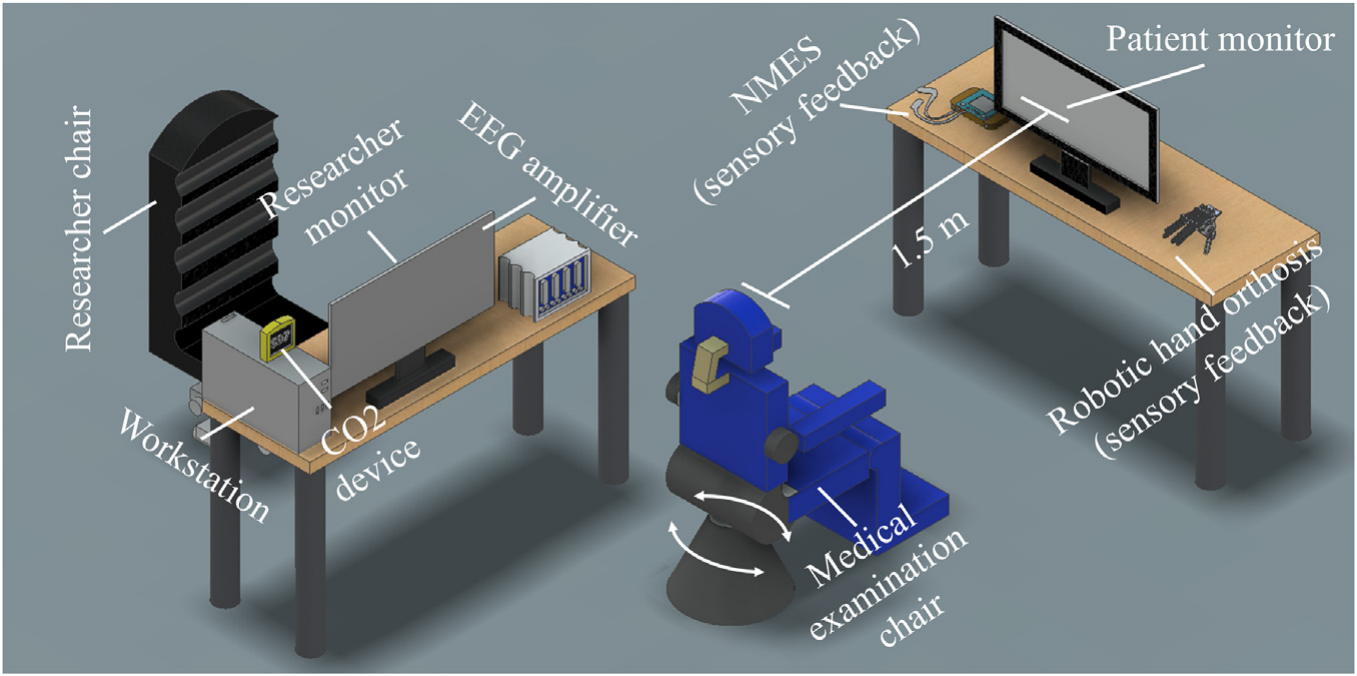
Setup for stroke neurorehabilitation interventions using a Brain-Computer Interface (BCI).
• *Medical examination armchair:* An electromechanical armchair with adjustable positions is ideal for the patient setup. It should include features such as seat rotation, vertical movement, and back tilt, as these adjustments are crucial for accommodating patients with hemiparesis and preventing fatigue associated with prolonged sitting.
• *Target for movement intention task:* A designated target, such as a baseball or a therapy ball, attached to one of the arms of the examination chair, is required for the movement intention task.
• *Therapeutic wrap:* This wrap is necessary for providing therapeutic support during the rehabilitation sessions.
• *Computer monitor:* A computer monitor with a screen size of at least 20 inches is recommended for displaying visual cues, stimuli, or feedback to the patient (referred to as the patient monitor).
• *Loudspeaker:* A loudspeaker, preferably attached to the patient's monitor, is needed for delivering auditory cues or feedback during the intervention.
• *Carbon dioxide measurement device:* A device for measuring carbon dioxide levels should be available to monitor the environmental conditions and ensure proper ventilation within the recording room.

• *Recording room:* A recording room of at least 5 m^2^ is recommended, equipped with sound-attenuated walls and proper external ventilation. It is preferable to have at least one window to ensure natural lighting (refer to Fig. 1 for an illustration of the recording room setup).

### EEG-based BCI System

• *BCI acquisition stage:* The EEG acquisition stage requires an EEG amplifier with a minimum resolution of 16 bits (although 24 bits is recommended), a sampling rate of at least 256 Hz, simultaneous acquisition capability for at least 8 channels (higher channel count provides better spatial resolution), and wired or wireless communication capability with a PC. Suitable systems meeting these specifications include g.USBAMP, g.NAUTILUS, g.HIAMP from g.tec, actiCHamp from Brain Products, or eego™mylab from ANT Neuro.
• *EEG electrode caps:* EEG electrode caps in small, medium, and large sizes are required. These caps should have marked positions based on the international 10–10 system and use active wet electrodes. Active wet electrodes contain built-in circuitry that improves the signal-to-noise ratio and effectively reduces motion artefacts within the EEG.
• *Workstation:* A workstation with at least an 8-core processor, 16GB RAM, and a discrete graphics card with two video outputs (e.g., DisplayPort or HDMI) is necessary. This workstation will handle the BCI processing stage.
• *Software:* Custom software with a graphical user interface (GUI) is typically used for the BCI processing stage. Existing commercially available software may not meet the requirements for BCI-based stroke interventions. The custom software, often programmed in MATLAB, C, or Python, enables the BCI operator to initiate the BCI intervention, calibrate the processing stage, control EEG amplifier acquisition, apply temporal filters, use feature extraction algorithms (e.g., FBCSP is recommended), employ a classifier algorithm (e.g., LDA is recommended), provide guided visual and auditory cues to the patient monitor and speaker, and maintain wired or wireless communication with the external feedback device. The GUI should also allow real-time visualization of the EEG signal, evaluation of correct EEG acquisition throughout the intervention, and measurement of electrode impedance.
• *Augmented feedback modality:* A specific augmented feedback modality is required to provide patients with awareness of their performance using the BCI system. This feedback can be visual, sensory, or a combination of both [9]. Visual feedback may involve a computer-generated hand replicating the target MI tasks displayed on the monitor. Sensory feedback can be provided through a robotic rehabilitation device, such as the commercial AMADEO system from Tyromotion, which enables finger movement through actuators and magnets [29,30]. Alternatively, researchers can design and build their own customized robotic assistive devices [24,25,31,32] that safely provide passive movement to spastic limbs. Another sensory feedback option is neuromuscular electrical stimulation (NMES), with systems like MotionStim from Medel GmbH being previously used in BCI studies [33].

### Clinical Measurements

**Fig. 2:**
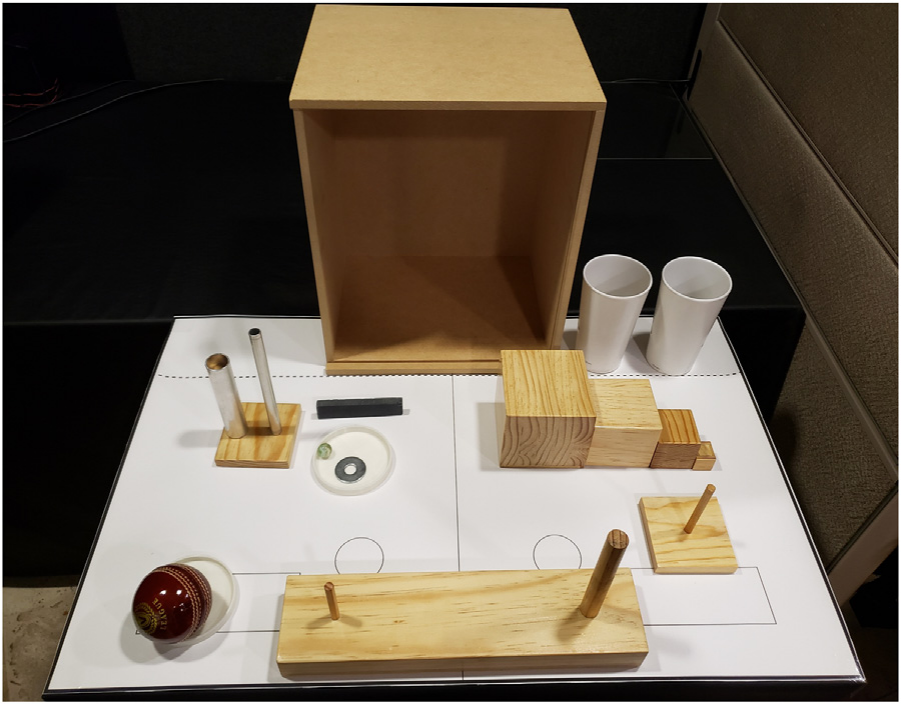
Items used in the Action Research Arm Test (ARAT) to assess upper extremity function in stroke.

• For the assessment of the upper extremity, the Fugl-Meyer Assessment for the Upper Extremity (FMA-UE) and the Action Research Arm Test (ARAT) are utilized. The FMA-UE provides a comprehensive evaluation of motor impairment, while the ARAT measures functional ability and dexterity. A standardized approach for administering the FMA can be found in the work by See et al., which includes scoring sheets and other relevant materials [34]. The ARAT requires specific items for its application, and the procedure for administering the test can be found in the study by Yozbatiran et al. [35]. Fig. 2 presents the items used in the ARAT.

## Procedures

### Patient Setup

• Assist the patient in sitting comfortably in the medical examination chair. Adjust the chair's position to ensure the patient's forehead is approximately 1.5 m away from the patient monitor.
• Clearly explain the procedure to the patient, providing detailed instructions. If necessary, rehearse the instructions to ensure comprehension, especially for first-time sessions or when previous difficulties in following the instructions were encountered.
• If the patient exhibits severe spasticity, secure the target limb with a therapeutic wrap.
• Minimize ambient noise and external distractions in the intervention room before commencing the session.
• Position the CO_2_ device within the researcher's sight but out of the patient's view.
• To mitigate the risk of airborne disease transmission, ensure proper ventilation by maintaining CO2 levels below 800 ppm in the intervention room [36].
• Attach the feedback device to the patient's limb for sensory feedback. If utilizing a robotic rehabilitation device, securely place and fasten it, verifying the correct execution of the desired passive movement during a test session. For NMES, position the stimulation electrodes on the desired locations and calibrate the amplitude accordingly. For hand grasping movements, electrodes can be placed surrounding the median nerve motor point (cathode) and on the forearm flexor muscular bellies (anode) to produce finger flexion; and on the radial nerve motor point (cathode) and on the forearm extensor muscular bellies (anode) to produce finger extension. An amplitude ranging from 5 mA to 20 mA and a rectangular biphasic pulse of 30 Hz with a duration of 300 µs can be used [37].

### EEG-based Bci System

**Fig. 3:**
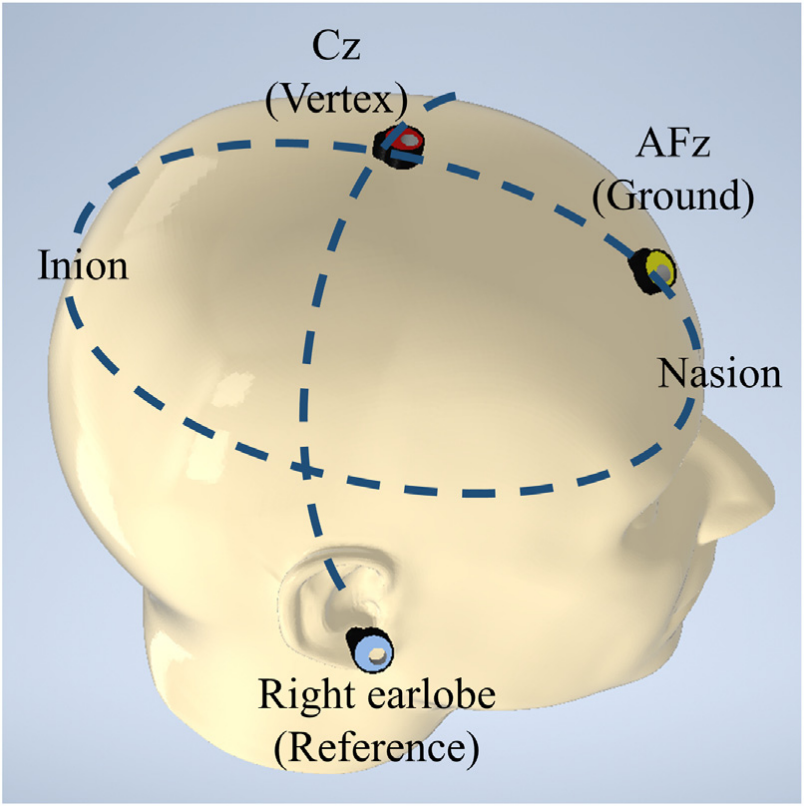
Anatomical reference points for electroencephalography (EEG) electrode placement.
• Fill the electrodes with gel, starting with the reference electrode, typically located on the right earlobe, and proceed from the electrodes closest to the right ear to those closest to the left ear until all electrodes are filled with gel.
• Power on the EEG amplifier and initiate EEG acquisition. Additionally, ensure that the preamplifier, if active electrodes are used, is powered on.
• Instruct the patient to blink, clench their teeth, and turn their head while monitoring all EEG channels on the workstation GUI for motion artifacts caused by electrode disconnections. Then, instruct the patient to close their eyes and verify the presence of alpha waves. Next, instruct the patient to open their eyes and confirm a decrease in the amplitude of alpha waves. Check the impedance of each EEG channel and ensure that it is below 5 kΩ.
• Each therapy session should consist of a minimum of 45 min of high-intensity therapy, as suggested by Langhorne et al. [38] who concluded that this duration is necessary for motor recovery of the upper and lower limbs in stroke patients. Although the number of sessions required for most patients to achieve minimal clinically significant recovery is still a topic of debate, studies have indicated that between 10 and 60 therapy sessions may be necessary to reach this rehabilitation threshold [39], [40], [41]. Additionally, each session should include the same number of runs and trials. A run is defined as a sequence of trials, and each trial comprises a complete set of tasks for the patient to perform. Within a 45-minute intervention session, it is feasible to complete 4 runs, each consisting of 20 trials. A trial should adhere to a predefined time structure, following the Graz paradigm [42]. An example of a trial structure for stroke patients can be seen in Fig. 4, as described in previous works [32]. Depending on the BCI transfer learning strategy employed, the processing stage of the system can be calibrated using runs from the current session or runs from previous session, which is feasible in stroke interventions [32].

• The EEG electrode cap should be positioned onto the patient's head. Identify the nasion, inion, and left and right preauricular points. Ensure that the Cz electrode is positioned at the vertex, where the vertical line connecting nasion and inion intersects with the horizontal line connecting the left and right preauricular points, as depicted in Fig. 3. A tape measure can be used to confirm that the distances between the Cz electrode and nasion/inion, as well as the Cz electrode and preauricular points, are equal.

**Fig. 4:**
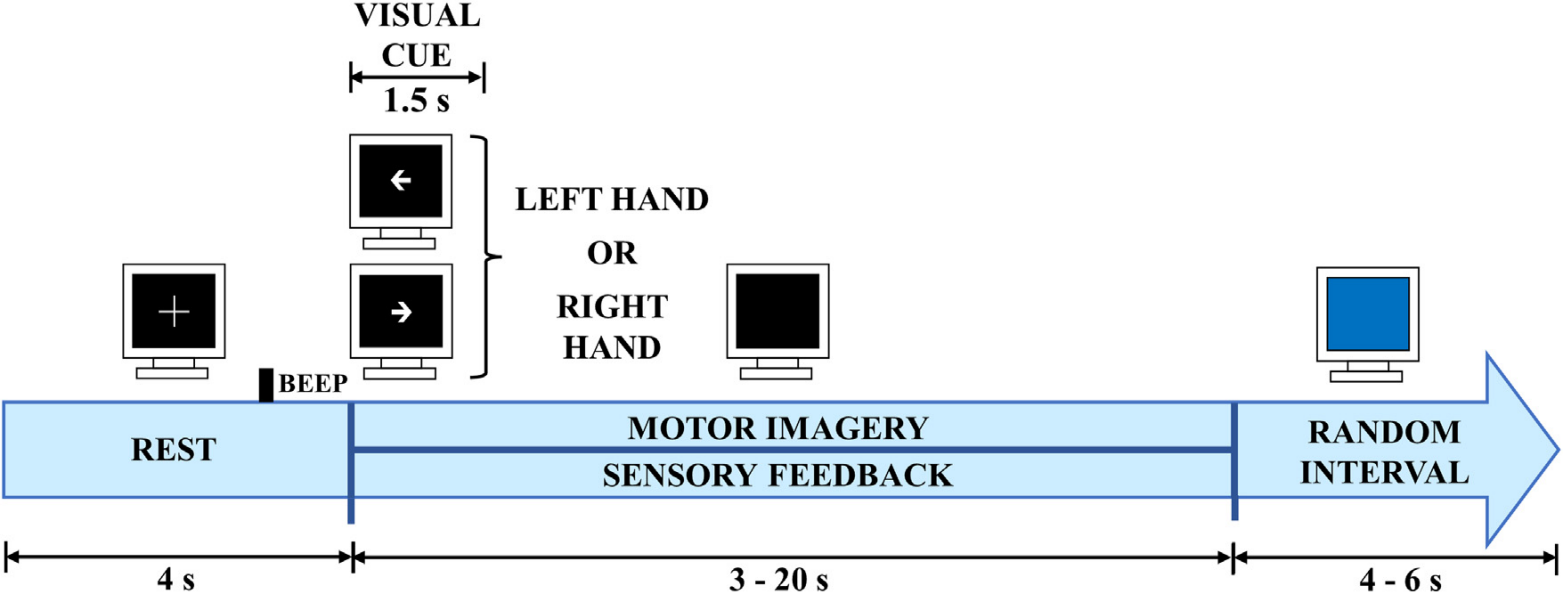
Example of a time structure for a trial utilized in a brain-computer interface (BCI) intervention for stroke.
• Initiate the BCI intervention within the GUI of the BCI software. Ensure that the EEG amplifier and any preamplifier (if active electrodes are used) are powered on.
• If the patient does not perform the tasks correctly, gently guide and correct them, preferably during the trial's rest and movement intervals to prevent artefact contamination of the EEG signal.
• If the patient is experiencing difficulties or achieving a low level of BCI control, advise them to remain calm and concentrate on the motor imagery (MI) task.
• After each run, inform the patient of the success rate of BCI control and provide instructions to help improve the execution of the MI task.
• After the intervention, inform the patient of the overall success rate of BCI control and offer recommendations to enhance the performance and execution of the MI task in the next session. Retrain the BCI processing stage using the new data from the current session if a "last session" transfer learning strategy is being employed.

### Clinical Measurements

• Conduct a pre-intervention measurement of the selected clinical tests before the start of the intervention, as well as a post-intervention measurement after the completion of the sessions. If the intervention consists of 30 or more sessions, additional measurements can be taken throughout the intervention. It is important to assign an experienced professional who can dedicate sufficient time to perform the measurements, as each test may take up to 30 min to administer.

## Observations

1. The graphical user interface (GUI) of the BCI software should be designed to be user-friendly, allowing non-engineering personnel to operate it. However, it is recommended to have at least one researcher or clinician with sufficient engineering background or training, present during the intervention, to address any issues that may arise.
2. Increasing the number of electrodes in the BCI setup can potentially improve BCI performance by providing higher spatial resolution. However, it can also extend the duration of the BCI intervention and increase the likelihood of encountering problems with EEG electrodes, such as losing contact with the scalp, which can lead to a decrease in BCI control. Using sixteen electrodes over the sensorimotor cortex strikes a good balance between spatial resolution and intervention setup time. The setup time for the BCI system should not exceed 15 min to avoid adding additional physical demand on the patients.
3. It is advisable to have the same team, consisting of a researcher and BCI operator, responsible for all BCI intervention sessions for a particular patient. This ensures consistency and familiarity throughout the intervention process.
4. Avoid conducting BCI sessions on patients with wet or recently dyed hair, as this can interfere with the impedance measurement of the EEG electrodes. In the case of recently dyed hair, it may also permanently stain the EEG cap.
5. Make efforts to maintain a positive mood among the patients. Stroke is often associated with depression, which can have a negative impact on intervention outcomes [43]. Actively listen to patients' needs and engage in conversations beyond the intervention instructions to create a supportive environment.
6. Stroke patients may suffer from aphasia or cognitive issues. It is important to assess these conditions to ensure that they are not severe, as this will help maintain adherence to therapy, which is a major determinant of treatment success.
7. Informing the primary caregiver of therapy advances, such as mentioning the patient's performance, the difficulties of using this type of systems and the progress made by the patient, also helps adherence to treatment.
8. Event-related desynchronization patterns in the time and frequency domains can be analyzed at the end of each session to assess whether the patient successfully performed the target MI tasks during the session trials.
9. If samples are missed during the analog-to-digital conversion of the amplifier/digitizer during EEG acquisition, it could be due to overlapping time required for sampling an EEG time window and processing that time window. To address this issue, consider optimizing the computational time required for BCI processing by refining feature extraction or classification algorithms, or by utilizing computational resources with faster processors.
10. During a BCI intervention, it is important to consider that patients' spasticity levels may change. In such cases, adjustments to sensory feedback parameters are necessary. These modifications may involve altering the torque or speed of a robotic assistance device or adjusting the amplitude of the electrical current or frequency used in NMES.
11. Each stroke lesion is unique, and therefore, the degree of recovery expected from a BCI intervention can vary. The extent of recovery appears to be primarily influenced by the amount of residual corticospinal tract tissue. Transcranial magnetic stimulation (TMS) can provide some insight into the level of residual tissue [44]. Patients who exhibit no voluntary residual movement in the affected limb are likely to have severe corticospinal damage and may have less pronounced clinically measurable improvements following BCI treatment [45]. However, it is worth noting that even under these circumstances, some patients can still achieve meaningful recovery, which holds significant clinical value [32].
12. TMS measurements serve as valuable secondary outcomes in BCI interventions for stroke, as changes in corticospinal excitability and integrity are associated with better primary outcomes [46]. Therefore, whenever feasible, incorporating TMS studies as complementary evaluations of the intervention is recommended.
13. BCI stroke interventions present an opportunity to collect EEG and other physiological measurements over several days. This data can be utilized to investigate neuroplasticity-related changes or to assess recovery prognosis [47,48].
14. The association between BCI performance and recovery in stroke is still not fully understood. Therefore, the primary measure of a BCI intervention's effectiveness should always be the clinically assessed recovery. Additionally, incorporating the patients' perspective as a secondary outcome can provide valuable insights.
15. It is important to note that BCI interventions are not solely beneficial for elderly stroke patients. Promising recoveries have also been observed in younger patients [49]. Furthermore, considering the reported association between COVID-19 and an increased risk of stroke [50], there is a likelihood of a rise in the number of young patients who could benefit from BCI rehabilitation, making it an encouraging approach for this specific demographic population.
16. The algorithms used for feature extraction and classification in the BCI processing stage must undergo prior validation for recognizing motor imagery (MI) in stroke patients. Examples of such validated algorithms include FBCSP, CSP, and LDA. If novel algorithms are planned to be employed, they should first be validated through offline and online non-interventional studies.

## Ethics Statements

Does not apply.

## CRediT authorship contribution statement

**Jessica Cantillo-Negrete:** Conceptualization, Methodology, Software, Writing – original draft, Data curation, Resources. **Ruben I. Carino-Escobar:** Conceptualization, Methodology, Software, Writing – original draft, Data curation. **Emmanuel Ortega-Robles:** Visualization, Investigation, Writing – review & editing. **Oscar Arias-Carrión:** Conceptualization, Writing – review & editing, Supervision, Project administration.

## Declaration of Competing Interests

The authors declare that they have no known competing financial interests or personal relationships that could have appeared to influence the work reported in this paper.

## Data Availability

No data was used for the research described in the article.
